# Using pH-Activable Carbon Nanoparticles as Cell Imaging Probes

**DOI:** 10.3390/mi10090568

**Published:** 2019-08-28

**Authors:** Honggui Lin, Jianlong Su, Ranjith Kumar Kankala, Mingrong Zeng, Shu-Feng Zhou, Xuexia Lin

**Affiliations:** 1 Department of Chemical Engineering & Pharmaceutical Engineering, College of Chemical Engineering, Huaqiao University, Xiamen 361021, China; 2 Provincial Key Laboratory of Naval Architecture & Ocean Engineering, Marine Engineering College Jimei University, Jimei University, Xiamen 361021, China

**Keywords:** carbon nanoparticles, cell imaging, pH responsiveness, cellular internalization

## Abstract

Herein, we demonstrate the fabrication of innovative pH-activable carbon nanoparticles (CNPs) based on urea and citric acid by microwave-assisted green synthesis for application in cell imaging. These CNP-based nanoprobes offer significant advantages of pH responsiveness and excellent biocompatibility. The pH responsiveness ranges from 1.0 to 4.6 and the slightly pH responsiveness ranges from 4.6 to 9.0. In addition, the pH-dependent modification of charge as well as the final diameter of the designed CNPs not only provide support as stable sensors for cell imaging under pH values from 4.6 to 9.0, but can also observe the pH change in cells from 1.0 to 4.6. Importantly, this significantly enhances the cellular internalization process resulting in tumor cell death. Together, we believe that these superior photoluminescence properties of our designed nanomaterials potentially allow for biological labeling, bioimaging, and drug delivery applications.

## 1. Introduction

Currently, most of the available cellular imaging probes are based on small molecules due to their low target-to-background ratios and excellent light-induced activation [1,2]. However, the applicability of small, molecule-based imaging probes is often hampered due to various disadvantages, such as the light-induced generation of free radicals resulting in toxicity issues and significant background autofluorescence during imaging, leading to reduced efficiency. To overcome this problem, it is required to fabricate a new fluorescent probe with low toxicity and high spatial–temporal resolution. As a critical physiological parameter, the pH value of the biological microenvironment plays a crucial role in cellular and tissue homeostasis on the activity of enzymes and microorganisms and most of the physiologically relevant pH ranges from 4.4 to 7.8 [3,4,5]. Numerous efforts have been dedicated to the development of pH-sensitive imaging probes with excellent specificity and sensitivity. In this framework, Urano and coworkers developed a pH-activable fluorescence macromolecule probe that could be activated after cellular internalization due to the rapid shift in the pH value in the lysosomes [6]. Jana and colleagues successfully synthesized red fluorescent CNP-based cell imaging functional nanoparticles using a high-temperature colloid-chemical approach coated with an amphiphilic polymer [7]. However, some acid-resistant cells require pH values below 1.8 [8,9], but it is very difficult for a normal pH probe to accurately detect pH values and cell imaging when the pH value is lower than 4.5. Furthermore, some acidic organelles also require a low acidic environment to maintain their function. For example, low pH is necessary for maintaining the digestive enzymes of lysosome (pH ranging 4.0 to 5.5) [10]. Therefore, it is necessary to develop a low pH-sensitive photoluminescent probe for cell imaging.

Photoluminescent carbon nanomaterials have been widely used in optical sensing applications [11], drug delivery [12], and cellular biology [13], among others [14]. Owing to their advantageous features of biocompatibility, environmental friendliness, and simple preparation methods, carbon-based photoluminescent nanomaterials have garnered enormous interest for application in biological labeling, bioimaging, and bioanalytical analysis [15,16,17]. These excellent properties of the carbon nanoparticles (CNPs) allow them to be considered as a useful alternative for a cell imaging application. However, the internalization of nanoparticles in cells often results in a significant change in their properties, which play a crucial role in the physiological functions [18]. Moreover, the uptake efficiency of differently charged nanoparticles is different in different cells [19,20]. Oftentimes, the internalization efficiency of positively charged nanoparticles is significantly higher in cells than the negatively charged species, while the internalized negatively charged nanoparticles can interact at a comparatively higher rate with nuclei, whose pH is reliably 0.3 to 0.5 units more than that of cytosol. In an attempt to demonstrate this fact, Liu and colleagues fabricated charge-switching nanoparticles that switch to positively charged under slightly acidic conditions and significantly facilitated their interactions with the negatively charged cell membranes to enhance their cellular uptake [21]. Further, they extended the design by using 2,3-dimethylmaleic anhydride (DMMA) to obtain a pH-dependent, charge-switchable polymer as an anticancer drug carrier. 

Motivated by these facts, we prepared environmentally friendly, pH-responsive, water-soluble CNPs for cellular imaging applications. The most interesting facts of the design are that the photoluminescence property of CNPs significantly increased with the increase of pH value of the surrounding microenvironment, and the prepared CNPs with negatively charged functional groups showed high resistance to non-specific protein adsorption, thereby exhibiting prolonged and stable luminescence. The prepared CNPs remarkably increased cellular internalization efficiency in vitro under weakly acidic conditions (pH 6.8) but were easily accumulated and internalized in a physiological environment. Furthermore, utilizing the intrinsic optical properties of CNPs, our CNPs could be used as pH-responsive sensors that would allow for cell imaging. 

## 2. Materials and Methods 

### 2.1. Materials

Urea, citric acid, and hydrochloric acid were purchased from Sinopharm Group Chemical Reagent (Shanghai, China). Cell counting kit 8 (CCK-8), Hoechst 33342, propidium iodide (PI), trypsin, penicillin/streptomycin, and phosphate buffer saline (PBS; pH 7.4) were obtained from Solarbio (Beijing, China). Calcein acetoxymethyl (AM) was obtained from Sigma-Aldrich (St. Louis, MO, USA). Dulbecco’s modified Eagle’s medium (DMEM) and RPMI-1640 medium were purchased from Yuanpei Biotechnology Co., Ltd. (Shanghai, China). Fetal bovine serum (FBS) was purchased from PAN Seratech (Leica TCS SP8, Wetzlar, Germany). 

### 2.2. Preparation of CNPs

The CNPs were synthesized using microwave-assisted synthesis following the reported procedure [11,22]. Briefly, citric acid monohydrate (6 g) and urea (6 g) were dissolved by ultrasonication in 20 mL of water. Then, the clear, colorless solution was heated in a microwave oven (Midea, Guangdong, China) for 5 min at 800 W, resulting in a brown, foamy solid. After cooling at room temperature, the brown precipitate was dissolved in 20 mL of ultrapure water, and the reaction mixture was centrifuged at 10,000 rpm for 20 min to remove the large-sized particles and unreacted substances. The resultant supernatant was further purified by dialysis for 3 days (membrane cutoff of the dialysis membrane was equivalent to ~500 Mw). Finally, the CNPs were freeze-dried and suspended at a concentration of 1 mg/mL^−1^ for cellular and characterization studies. Further, the fabricated CNPs were confirmed by measuring the fluorescence intensity under UV irradiation at 365 nm.

### 2.3. Characterizations

The ultraviolet–visible (UV-vis) absorption spectra of CNPs were recorded on a UV-vis spectrophotometer (UV-2600, Shimadzu, Tokyo, Japan). Fluorescence data were measured on a luminescence spectrometer (F-320, Gangdong Technology Co., Ltd., Guangdong, China). Fourier transform infrared (FT-IR) spectra were obtained on a Nicolet Is 50 Fourier-transform infrared spectroscopy (FTIR) spectrometer (Thermo, Waltham, MA, USA). The X-ray diffraction (XRD) patterns were recorded on a Smart La (Rigaku, Tokyo, Japan) operated at 30 mA and 40 KV using Cu Kα radiation scanning from 5° to 70° at a scan rate of 5° min^−1^. The morphologies of the samples were studied using a high-resolution transmission electron microscopy (Talos F200S, FEI Ltd., Hillsboro, OR, USA). The diameter and zeta potential (ζ) of the samples were determined using a Zetasizer Nano ZS (NanoBrook Omni, NY, USA). Images of fluorescence luminescence were obtained using a ZYF-2000E fluorescence microscope (Zhaoyi Optoelectronic Technology Co. Ltd., Shanghai, China). Cellular fluorescence imaging was carried out using a confocal laser scanning microscope (CLSM) (Leica TCS SP8, Wetzlar, Germany). The Raman spectra were recorded using a DXR microscope spectrometer (Thermo, Waltham, MA, USA) at a laser excitation line of 532 nm. 

### 2.4. Cell Studies In Vitro 

#### 2.4.1. Cell Culture 

Human umbilical vein endothelial cells (HUVECs from American Type Culture Collection (ATCC), Manassas, VA, USA) were cultured in Roswell Park Memorial Institute (RPMI)-1640 medium supplemented with 10% FBS and 1% penicillin/streptomycin. Hela cells (ATCC) were cultured in Dulbecco’s modified Eagle’s medium (DMEM) supplemented with 10% FBS and 1% penicillin/streptomycin and incubated in a humidified incubator (37 °C, 5% CO_2_). 

#### 2.4.2. In Vitro Cytotoxicity 

The cytotoxic effect of the CNPs was studied using the CCK-8. In brief, 100 μL of HUVECs and Hela cells were seeded in 96-well plates at a density of 5 × 10^3^ cells/mL. After 24 h, the culture medium was discarded and cells were treated with 150 μL of medium containing various concentrations of CNPs (1, 10, 20, 50, 100 μg/mL) adjusted to a pH value of 6.8 or 7.4 for 24 h, 48 h, and 72 h, along with the media as a negative control. At the predetermined intervals, the medium from each well was replaced with 100 μL of fresh medium containing CCK-8 working solution after the cells were washed twice with PBS. After 1 h, the viability of the cells was detected using a microplate reader at 450 nm. The experiment was carried out in triplicate. Further, the viability of Hela cells and HUVECs was qualitatively confirmed by live/dead assay kit using calcein AM and PI based on visualizing fluorescence using confocal laser scanning fluorescence microscopy (CLSM).

### 2.5. Cellular Imaging

Hela cells and HUVECs were seeded at a density of 1 × 10^4^ cells/mL in the CLSM dishes and cultured overnight. Further, the media was replaced by fresh media containing 10 μg/mL CNPs and then incubated for 2 and 6 h. Then, the medium was discarded and washed thrice with PBS buffer to remove the free CNPs. To study the pH-responsive CNPs for cell imaging and cellular cytotoxicity, the new medium containing CNPs was adjusted to a pH value of 6.8 or 7.4. Finally, the samples were observed using CLSM and excited by a 405 nm laser.

## 3. Results and Discussion

### 3.1. Characterization of CNPs 

**Figure 1:**
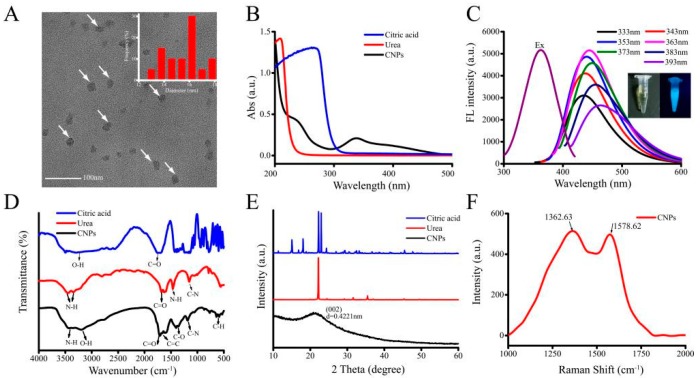
Physical characterization representing the morphology and chemical functionalities using various techniques. (**A**) High-resolution transmission electron microscopy (HR-TEM) image of carbon nanoparticles (CNPs). Inset showing the statistical analysis of the particle size distribution of CNPs. (**B**) The ultraviolet–visible (UV-vis) absorption spectra of CNPs, urea, and citric acid. (**C**) The fluorescence excitation spectra of CNPs and emission spectra of CNPs at the different excitation wavelengths (inset shows CNPs under visible light, and right shows CNPs excited in UV light). (**D**) Fourier-transform infrared spectroscopy (FTIR) spectra and (**E**) X-ray diffraction (XRD) patterns of CNPs, urea, and citric acid. (**F**) Raman spectrum of synthesized CNPs.

The surface morphology of CNPs from transmission electron microscope (TEM) images (Figure 1A) depicted that the average diameter of CNPs was in the range of 12 to 18 nm. From the UV-vis absorption spectra, the prepared CNPs (black) exhibited UV-vis absorption from 250 to 350 nm (it should be noted here that citric acid (blue) and urea (red) exhibit only UV absorption below 250 nm). It was also observed that the CNPs resulted in altered absorption peaks at around 233 and 337 nm, due to the π-π* transition of the C=C bond, and the n-π* transition of the C=O bond (Figure 1B), respectively, which confirms that the sp^2^ clusters were contained in CNPs. As depicted in Figure 1C, the fluorescence spectra of the synthesized CNPs resulted in the maximum excitation located at 363 nm as well as emission wavelengths located at 444 nm. The differences in the spectra of the synthesized CNPs and CNPs under UV irradiation at 365 nm were presented in the inset of Figure 1C. The color of the resultant solution was changed to blue under UV irradiation (365 nm). Further, the luminescence peak gradually shifted to a longer wavelength with increasing excitation wavelength from 333 to 393 nm (Figure 1C), indicating a distribution of the different surface energy traps of the CNPs due to the abundant functional groups of CNPs, such as carboxylic acid, amine, and hydroxyl functional groups.

To further explore the chemical functionalities of CNPs, we used the FTIR spectroscopy for analysis (Figure 1D). The FTIR spectrums of urea (red) and citric acid (blue) showed their characteristic peaks, such as –NH_2,_ –CON–, and –COOH. The FTIR spectrum for the CNPs (black) is shown in Figure 1D. The peak at 1060 cm^−1^ could be attributed to the absorption band of the oxygen-containing core. The characteristic absorptions at 3427 cm^−1^ and 3350 cm^−1^ could be ascribed to the stretching vibration of passivated -NH_2_ molecules of urea. Moreover, the carboxyl groups could be explicitly identified by both O-H stretching at 3202 cm^−1^ and C=O stretching at 1710 cm^−1^. The peak at 1603 cm^−1^ and the absorption peaks at 870 cm^−1^ and 770 cm^−1^ could be attributed to the C=C stretching and C-H stretching of benzene. The absorption bands at 1399 cm^−1^ and 1366 cm^−1^, as well as 1060 cm^−1^, could be assigned to C-N stretching vibrations from aromatic amine and aliphatic amino groups, respectively. The results reveal that carboxyl, hydroxyl, amino, and amide groups exist on the surface of CNPs. These hydrophilic functional groups result in good dispersibility of CNPs in water. Raman spectroscopy (Figure 1F) of CNPs showed two broad peaks at around 1362.63 and 1578.62 cm^−1^, attributed to the D-band (sp^3^ hybridization) and G-band (sp^2^ hybridization). The D-band represents a vibration mode of the carbon atom and the G-band is associated with the vibration mode. The bands indicate that the CNPs are amorphous.

The crystalline nature of CNPs, urea, and citric acid was further identified by XRD. As presented in Figure 1E, the raw urea and citric acid were highly crystalline materials, however the CNPs have a broad peak at 21° that is attributed to the graphitic structure with interlayer spacing (002) of 0.4221 nm, corresponding to typically amorphous carbonaceous materials compared with raw materials.

### 3.2. pH-Sensitive Fluorescence Intensity of the CNPs

**Figure 2:**
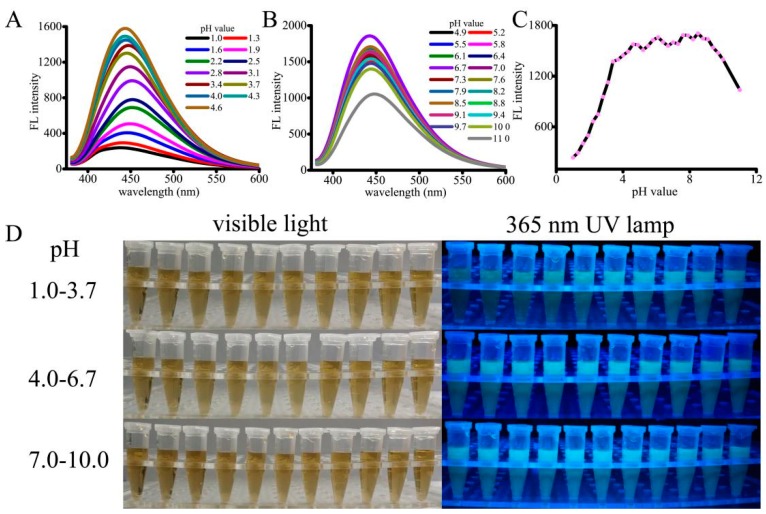
In vitro pH-dependent fluorescence and luminescence behaviors of CNPs: pH-sensitive fluorescence spectra of the CNPs, ranging from pH 1.0 to 4.6 (**A**) and 4.9 to 11.0 (**B**) at 363 nm excitation; (**C**) pH-sensitive fluorescence intensity of the CNPs with respect to change in pH value. (**D**) The photographs of CNP solution under visible light and a 365 nm UV lamp.

The fluorescence as well as luminescence behaviors of the pH-sensitive CNPs were investigated at various pH values. As shown in Figure 2, the fluorescence intensity was significantly changed with the increase of pH from low to high. Under strongly acidic or alkaline conditions, the photoluminescence was very weak, whereas the CNPs showed the strongest emission intensity at the solution pH value of 4.6 (Figure 2A,C). Interestingly, the fluorescence intensity of CNPs was increased significantly in the pH values ranging from 1.0 to 3.4, and the increment was slightly slow from pH values of 3.4 to 4.6. From Figure 2B,C, it is observed that the intensity was reduced rapidly at pH values higher than 9.1. These observations indicate that the CNPs would possess strong acidic sites relevant to the luminescence emission. Further, the effect of pH responsiveness was confirmed by adjusting the pH value back to the range of 4.6 to 9.1, where the fluorescence intensity of the CNPs befitted high substantially. Such a pH range (4.6–9.0) is similar to physiological pH environments or tumor pH environments (normal pH 7–8, tumor pH ≤ 7; lysosomal pH values can be as low as 4.0–4.5). Plausibly, the reversible protonation of CNPs might be the reason for the variation of the luminescent intensity. The photographs of the CNP (10 μg/mL) solution under visible light and a 365 nm UV lamp are shown in Figure 2D. Together, these results demonstrate that CNPs with excellent fluorescence properties could be used as pH sensors and have potential applications in cell imaging.

### 3.3. pH-Responsive Changes in the Surface Charge as Well as the Diameter of CNPs

**Figure 3:**
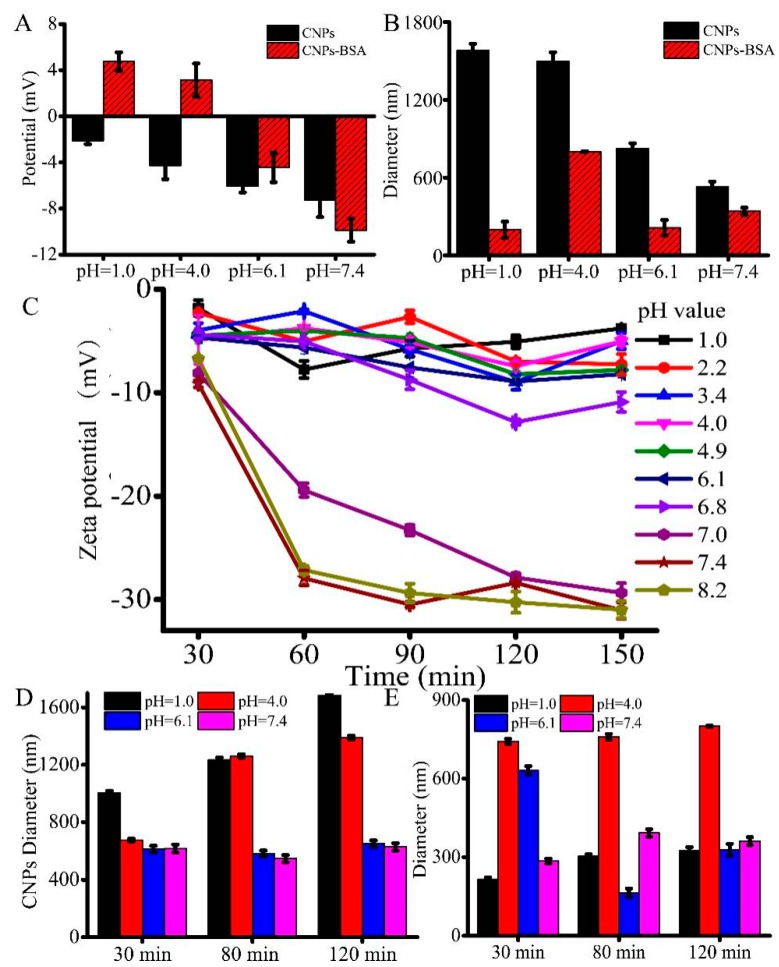
Effect of pH on the particle diameter as well as zeta potential of CNPs: (**A**) pH effect on the potential of the CNPs; (**B**) pH effect on the diameters of the CNPs. (**C**) Time-dependent zeta potential of CNPs by incubation at different pH values. (**D**) Time-dependent diameter changes of the CNPs by incubation at different pH values. (**E**) Time-dependent diameter changes of the CNP-BSA solution by incubation at different pH values.

Further, we investigated the pH-responsive changes in the overall surface charge of CNPs at pH values of 1.0, 4.0, 6.1, and 7.4, in which the pH of 6.1 is close to the tumor extracellular environmental pH, while 7.4 represents the pH value of the normal physiological environment. As the designed CNPs are sensitive in the pH range of 1.0 to 4.6, we also investigated the changes in the potential of the CNPs in that particular pH range as well. It was observed from the results that the potential of the CNPs was gradually decreased, showing a substantial charge conversion with the increase of pH values, indicating that the CNPs have shown substantial sensitivity to the change of pH value. In addition, an unstable negative charge at pH values higher than pH 1.0 was shown (Figure 3A). Meanwhile, the gradual decrease in the sizes of CNPs was observed with the increase of the pH values, indicating that the amide bonds would be cleaved under alkaline conditions or weak acid conditions, leading to the alkaline-induced detachment of these nanoparticles. To further demonstrate whether the CNPs could combine with the proteins, CNPs were mixed with 1% bovine serum albumin (BSA) solution and investigated the changes in the potential of the eventual construct were investigated. It was observed that the potential of the CNP solution remained positive at a pH value lower than 4.0, while it was negative at pH values higher than pH 4.0. This pH-responsive charge reversibility indicates that these CNPs could be used for cell imaging applications. On the other hand, although the potential of CNP-BSA is sensitive to the pH value, the same tendency in the case of CNP-BSA sizes was not observed. BSA was adsorbed onto the surface of CNPs by reducing the alkaline-induced interactions, which were relatively weaker and resulted in stable CNP-BSA (Figure 3B). As shown in Figure 3B, it can be easily found that the diameter of CNPs is larger than that of CNP-BSA. We deduced the hydration functions of CNPs and CNP-BSA. CNPs have a much stronger hydration function and larger hydration size than that of CNP-BSA, leading to decreased particle sizes of CNP-BSA. Moreover, although BSA adsorption on the surface increases opsonization, which is not a desirable quality, it promotes convertible charge (Figure 3A) and then it provides the possibility of cellular internalization of CNPs. To further understand the charge changes of CNPs under different pH values, the hydrodynamic potential of CNPs was also detected (Figure 3C). A slight decrease in the potential of CNPs was observed, indicating that the fluctuation of the potential was stable after 60 min (Figure 3C). Some significant tendencies towards a decrease in the negative zeta potentials of CNPs were observed, while the pH value tended to be weakly acidic or alkaline (pH ≥ 6.1), demonstrating the alkalescent, environment-induced detachment of CNPs. 

The DLS measurements of CNPs show that the changes in the hydrodynamic sizes of CNPs were unstable before 110 min (Figure 3D), while the hydrodynamic sizes of CNPs with BSA solution were relatively stable with the increase of time (Figure 3E). A significant increase in the diameter of CNPs was observed for CNPs at pH ≤ 4.0, but not for the same sample at pH 7.4, indicating the acid-induced aggregation and the alkaline-induced detachment behaviors of CNPs. Nevertheless, the CNP-BSA solution at pH 4.0 showed the largest diameter, which gradually decreased to both sides. Together, the experimental data demonstrated that proteins can be absorbed on the surface of CNPs, and would protect the detachment of CNPs. Moreover, these evidences of protein attachment could also prove that CNPs can substantially enter the cells.

### 3.4. Cytotoxicity of CNPs

**Figure 4:**
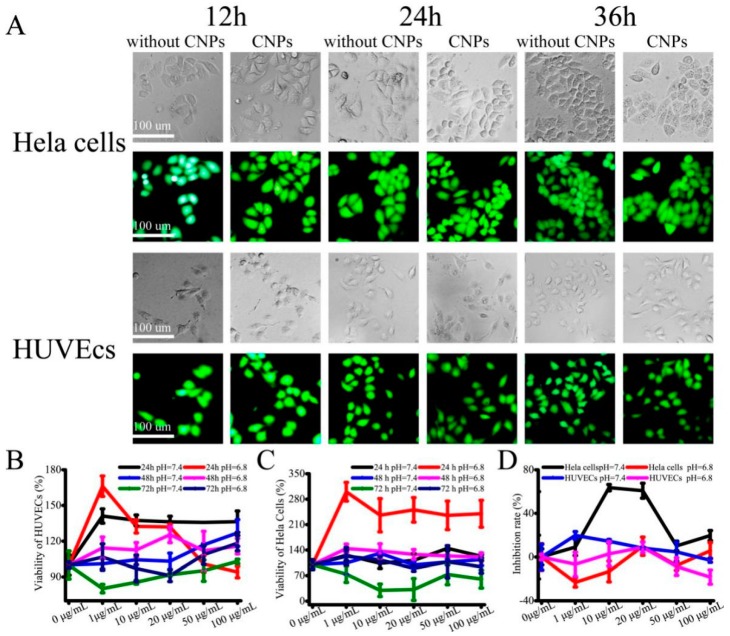
Cytotoxicity in vitro of designed CNPs. (**A**) Fluorescent images of Hela cells and HUVECs (stained with calcein-AM/PI) treated with 10 μg/mL CNPs. Time-dependent viability of cultured HUVECs (**B**) and Hela cells (**C**) treated with CNPs at different concentrations. (**D**) Concentration-dependent cell inhibition.

The cytotoxicity effects of CNPs were investigated by quantitatively determining the dead and live cells using calcein-AM and PI in Hela cells and HUVECs. Hela cells and HUVECs were cultured with 10 μg/mL CNPs added to the medium. During 36 h of treatment, the amount of green fluorescence (live cell staining) was significantly higher in Hela cells and HUVECs compared to the red fluorescence (Figure 4A). These results clearly demonstrate that the designed CNPs were highly compatible. We then evaluated the cytotoxicity of the CNPs in cultured HUVECs and Hela cells by spiking CNPs (0–100 μg/mL) into the cell culture medium. This showed that the developed CNPs have very different cytotoxicity for cultured HUVECs over three days because the viability of HUVECs determined by the CCK-8 assay was still as high as 80% (Figure 4B). However, after Hela cells were incubated with CNPs for three days (Figure 4B), the viability of the Hela cells reduced to 30% (Figure 4C). These results indicated that CNPs have different cytotoxicity for different kinds of cell lines. The inhibitory concentration (IC50) of CNPs against cultured Hela cells for 72 h was also calculated (7.98 μg/mL; Figure 4D). These data implied that the CNPs have little effect on the viability of HUVECs, but they have shown considerable toxicity on Hela cells. We speculate that this is mainly due to the heterogeneity of cells and differences in cell uptake of the nanoparticles [23,24,25,26,27]. Thus, the different viability of Hela cells was observed. Moreover, it was also evident that the cells cultured in pH 6.8 medium had higher viability than that in the pH 7.4 medium. We deduced that the alkaline environment made the particle diameter of CNPs smaller, which is beneficial to the uptake of CNPs into cells, resulting in the enhanced toxicity. 

### 3.5. Cell Imaging 

**Figure 5:**
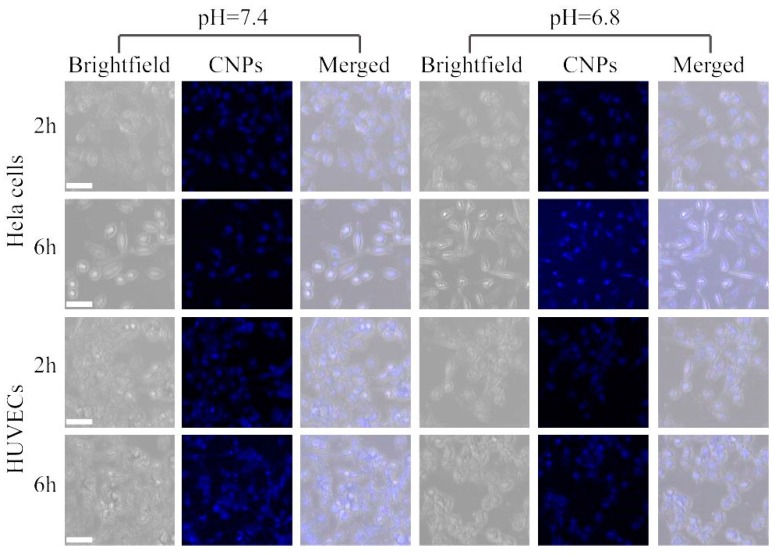
Confocal images of Hela cells and HUVECs after incubation with 10 μg/mL CNPs for 2 and 6 h at 37 °C. All scale bars are 50 μm.

As is well known, pH plays a critical role in cellular and tissue homeostasis, and abnormal pH is related to many major diseases, such as Alzheimer’s disease. In normal tissue, the interstitial pH is carefully maintained at 7.2–7.4, whereas the extracellular pH of solid malignant tumors is acidic, in the range of 6.5–6.8. Using CNPs as a pH probe, the fluorescence intensities of the CNPs increased with increasing pH, while the intensity was only slightly changed ranging from pH 4.9 to 9.0 in Figure 2. This fluorescence spectral response of CNPs toward pH allowed us to quantify the signal using the fluorescence intensity. Furthermore, the fluorescence signal at pH 5.5 is 1.03-fold and 1.01-fold that of at pH 6.8 and pH 7.4, respectively. These experimental results indicate that this probe could effectively form a stable luminous signal without being affected by the pH of the microenvironment. As shown in Figure 5, Hela cells and HUVECs were incubated with CNPs at different pH levels of 6.8 and 7.4 for 2 h. Cellular imaging results showed high spatial resolution and low light scattering. In addition, a much stronger photoluminescence signal with CNPs inside cells in a pH 6.8 environment was observed compared to those in the pH 7.4 environment. A much stronger photoluminescence signal was observed together with CNPs inside Hela cells and HUVECs, including in the cytoplasm and nucleus, at pH 6.8, while there was only a slight photoluminescence signal with CNPs inside the cytoplasm of two kinds of cells at pH 6.8. Together, we demonstrate that the pH environment may affect the uptake of CNPs by cells and the changes of the photoluminescence signal would be due to cellular uptake of CNPs, as the fluorescence intensity has no notable effect on the pH of the environment (pH ranging from 4.6 to 9.0). However, it was observed that the photoluminescence signal of CNPs was enhanced in the nuclei of Hela cells and HUVECs but decreased in the cytoplasm after incubation for 6 hours. Furthermore, the decrease in the photoluminescence signal of the cytoplasm at pH 7.4 was much less than that at pH 6.8, meaning that the CNPs could be accumulated and internalized easier at pH 7.4. Furthermore, the nucleus showed a higher signal, indicating that many more CNPs could be accumulated and internalized. More importantly, Hela cells showed much higher photoluminescence signals than HUVECs, implying that CNPs could be easier accumulated and internalized in tumor cells. These experimental results suggest that our CNPs can be used as sensors for cell imaging and exhibit significant tumor cellular internalization. 

## 4. Conclusions

In this work, a simple approach was developed in synthesizing CNPs with low toxicity and high biocompatibility. The CNP solution showed outstanding optical stability and marked pH responsiveness. Furthermore, the CNPs could be applied to cellular imaging, showing high spatial resolution and low light scattering. Most importantly, the toxicity of CNPs was enhanced for tumor cells but not for normal cells, indicating that the CNPs have potential applications in biolabeling, bioimaging, and other pharmaceutical applications.
